# The MiRNA Journey from Theory to Practice as a CNS Biomarker

**DOI:** 10.3389/fgene.2016.00011

**Published:** 2016-02-09

**Authors:** Nicoleta Stoicea, Amy Du, D. Christie Lakis, Courtney Tipton, Carlos E. Arias-Morales, Sergio D. Bergese

**Affiliations:** ^1^Department of Anesthesiology, The Ohio State University Wexner Medical CenterColumbus, OH, USA; ^2^College of Medicine, The Ohio State UniversityColumbus, OH, USA; ^3^Department of Neurological Surgery, The Ohio State University Wexner Medical CenterColumbus, OH, USA

**Keywords:** MicroRNA, biomarker, cerebrospinal fluid (CSF), central nervous system (CNS), neurodegenerative disease

## Abstract

MicroRNAs (miRNAs), small nucleotide sequences that control gene transcription, have the potential to serve an expanded function as indicators in the diagnosis and progression of neurological disorders. Studies involving debilitating neurological diseases such as, Alzheimer's disease, multiple sclerosis, traumatic brain injuries, Parkinson's disease and CNS tumors, already provide validation for their clinical diagnostic use. These small nucleotide sequences have several features, making them favorable candidates as biomarkers, including function in multiple tissues, stability in bodily fluids, a role in pathogenesis, and the ability to be detected early in the disease course. Cerebrospinal fluid, with its cell-free environment, collection process that minimizes tissue damage, and direct contact with the brain and spinal cord, is a promising source of miRNA in the diagnosis of many neurological disorders. Despite the advantages of miRNA analysis, current analytic technology is not yet affordable as a clinically viable diagnostic tool and requires standardization. The goal of this review is to explore the prospective use of CSF miRNA as a reliable and affordable biomarker for different neurological disorders.

## Introduction

MicroRNAs (miRNA) are small, noncoding RNA fragments approximately 22 nucleotides long involved in post-transcriptional regulation of gene expression (Guan et al., 2010; Gallego et al., 2012; Cheng et al., 2013). After processing by endonucleases (Cheng et al., 2013; Freischmidt et al., 2013), the resultant single-stranded miRNAs combine with other macromolecules to form RNA-induced silencing complexes (RISC). These RISCs target complementary messenger RNA (mRNA) strands for degradation, altering cellular function (Cheng et al., 2013; Freischmidt et al., 2013).

MiRNAs are thought to have widespread action as more than 1500 identified miRNAs have been shown to affect the expression of 30–60% or more of all genes (Siegel et al., 2012; Ksiazek-Winiarek et al., 2013; Sheinerman and Umansky, 2013; Cloutier et al., 2015). MiRNA plays a role in virtually all cellular functions, such as cell proliferation, differentiation, and importantly apoptosis (Guan et al., 2010; Siegel et al., 2012; Ksiazek-Winiarek et al., 2013). Additionally, miRNAs are widely present in all body tissues and fluids such as plasma, serum, urine, saliva, milk and CSF (Siegel et al., 2012; Galimberti et al., 2014).

Various miRNAs subtypes are differently expressed among the organs of the body and within the organs themselves. For example, a patient with Alzheimer's disease (AD) will express a different miRNA profile in the medial frontal gyrus, hippocampus, and cerebellum (Cogswell et al., 2008). Due to its pervasiveness, stability in bodily fluids, and dysregulation in early disease course, miRNA could be considered a reliable biomarker.

In our analysis, we explored the recent history of serum and CSF protein biomarkers, discussed the advantages of CSF miRNA, and examined the utility of CSF miRNA in CNS disease management.

## Methods

In assessing the reliability of miRNA as a biomarker, we conducted an extended review of literature on CSF miRNA in CNS disorders, published within the last 5 years. Choice landmark studies were also included.

### Serum and CSF protein biomarkers

Serum and CSF protein biomarkers are able to provide a fast and accurate diagnosis of various diseases. For example, CSF IL-8 and serum TNFα, are considered sensitive markers of neuroinflammation (Maxeiner et al., 2014). Furthermore, another well-studied CSF biomarker, IL-6, along with IL-8 have been found to be elevated in patients with depression and delirium (van Munster et al., 2008; van den Boogaard et al., 2011; Liu et al., 2013; Kern et al., 2014).

CSF and serum biomarkers also have shown predictive value in evaluating neurologic disease progression. Czeiter et al. demonstrated that, serum and CSF GFAP_24S levels facilitated traumatic brain injury prognosis alongside the IMPACT calculator (Czeiter et al., 2012). Several studies have also investigated AD biomarker prognostic potential, including the ability to track cognitive decline post-surgery and progression into AD (Ackermann et al., 2014). Two such CSF biomarkers, neurogranin and tryptic peptide SNAP-25, should be able to track AD progression. Furthermore, neurogranin specifically identifies MCI patients at risk for developing AD (Brinkmalm et al., 2014; Kvartsberg et al., 2014).

CSF protein biomarkers, α2HS glycoprotein, α1 antichymotrypsin, and α1beta glycoprotein, may predict the benefit of ventriculoperitoneal shunt for patients with normal pressure hydrocephalus (NPH) (Scollato et al., 2010). NPH patients with improved shunt response also have been linked to significantly lower CSF levels of sAPPα and sAPPβ (Pyykö et al., 2014).

Although these protein biomarkers have promising diagnostic, prognostic, and therapeutic benefits, recent literature explores their limitations. Interestingly, only 77.2% of 2357 AD patients expressed disease related CSF Aβ and either T-tau or P-tau protein values (Hoglund et al., 2015; Rosén et al., 2015). MiRNA appears to predict and diagnose AD with greater sensitivity, even during mild cognitive impairment. For example, Alexandrov et al. noticed miR-9, miR-125b, miR-146b, and miR-155 upregulation in the diseased state when no significant correlation was established between Aβ and AD (Alexandrov et al., 2012).

### Advantages of using miRNA as a biomarker

Because of its widespread action and presence, miRNA profile changes enable the identification of many pathological processes, including malignancies and cardiovascular and neurological diseases (Calin et al., 2002; Bonauer et al., 2010; Siegel et al., 2012).

MiRNAs' stability is a promising biomarker quality. Interestingly, Siegel demonstrated a small percent of circulating miRNAs are protected from degradation due to their inclusion in small vesicles known as exomes; furthermore, peripheral miRNAs included in RISC complexes avoid degradation by RNAses (Siegel et al., 2012). MiRNA is unique in playing a central role in gene regulation. Schramedei et al. found miR-21 to be a key oncogenic regulator, downregulating tumor suppressors ANP32A and SMARCA4 (Schramedei et al., 2011). Thus, miR-21 could be a more reliable indicator of disease progression than other tangential biomarkers in the progression of cancers, like CNS tumors. CSF elevation of let-7b, a miRNA precursor, is correlated with AD. When administered *in vitro*, let-7b was shown to proportionally decrease neuronal survival while sparing cell lines containing mutated TLR7 or downstream proteins. Lehmann et al. therefore concluded that let-7b causes neurodegeneration in AD through activation of Toll-like Receptor 7 (Lehmann et al., 2012). Ghidoni et al. linked a decreased miR-107 in early AD to BACE1, a pathogenic compound in AD (Ghidoni et al., 2011). Unfortunately AD clinical manifestations and post-mortem diagnosis often overlap with other forms of dementia such as frontotemporal or Lewy body dementia (Humpel, 2011). Moreover, observable symptoms manifest late in the clinical course impairing early diagnosis and prevention. Other potential biomarkers often appear in much later stages of the disease when neuronal damage has already occurred (Kim et al., 2014). Similar to AD, amyotrophic lateral sclerosis (ALS) identification also relies heavily on clinical presentation, responsible for misdiagnosis in 8–44% of cases (Cloutier et al., 2015).

### CSF as a source of miRNA

In patients with neurological disorders, CSF is the ideal source of miRNA, its profile being identical to that of the brain tissue because of its close proximity. CSF sampling from lumbar puncture is often standard procedure for the diagnosis of MS and ALS (Polman et al., 2005; The ALS Association, 2015). Even though tissue biopsy may be more accurate than CSF collection, CSF sampling has the benefit of being much less invasive.

### CSF processing

Method of extraction of CSF usually varies. As a standard procedure, after CSF is collected, centrifugation (500 × g, 10 min, and room temperature) needs to be performed within 60 min after collection in order to remove cells and debris. Storage of centrifuged samples is usually at −80°C. Baraniskin et al. (2011) MicroRNA can be extracted using different kits on the market, and manufacturer instructions have to be followed carefully in order to yield the maximum RNA possible. Although 1 mL of CSF only yields 15–30 ng of total RNA, Burgos et al. reported that is possible to obtain reproducible results from as little as 0.5 mL of human CSF (Burgos et al., 2013).

MiRNA can also be isolated from a multitude of blood sources, including serum, plasma, leukocytes, and whole blood samples, establishing its effectiveness as a biomarker (Cheng et al., 2013; De Felice et al., 2014). While both CSF and serum miRNA correlate well with many pathological processes, blood sources are often preferred over CSF sources because they are less invasive and accessible (De Felice et al., 2014). Within the context of neurological disorders, however, CSF typically yields more accurate results (Freischmidt et al., 2013; Cloutier et al., 2015). A meta-analysis examining data from over 20 original works on CNS malignancies found that CSF-based miRNA profile was more sensitive and specific than blood-derived miRNA. In this meta-analysis, the sensitivity for detecting CNS malignancies increased from 83% for blood-derived miRNA to 86% for CSF miRNA while the specificity increased from 81% in blood to 88% in CSF (Wei et al., 2015).

Blood volume is greater than CSF volume and thus, miRNA concentration is diluted when the nucleotide sequences cross from the CSF through the blood-brain barrier (BBB) and into the blood. This dilution process leads to less clarity in the diagnostic findings. Furthermore, while CSF contains miRNAs shed directly from the brain, blood contains miRNAs shed from all bodily tissues. For example, peripheral tissues excrete large amounts of miR-21 that can interfere with the diagnosis CNS tumors which also excrete miR-21 (Akers et al., 2013). In the case of miR-21 excreting CNS tumors, CSF sampling is therefore preferred. While certain miRNA exist in both blood and CSF, they are often poorly correlated. Furthermore, other miRNA subtypes have been exclusively identified in CSF or blood. Burgos et al. also compared microRNA present in serum and CSF from five subjects, and the study revealed that profiles of microRNA are more similar from different subjects for either CSF or serum and are less similar to one another, even within the same subject. The reasoning of this finding is due to microRNAs in CSF are derived from neural cells, whereas serum microRNAs are collected from all tissues in the body. The authors concluded based on the results, that while there are a large number of microRNA unique to CSF and serum, most miRNAs are present in both of them (Burgos et al., 2013). Also, sequencing small RNAs from small volumes can provide a large amount of reproducible data.

Early studies seem to suggest that the BBB plays a minor role in dictating the passage of miRNA between CSF and blood. The BBB becomes more permeable as a result of the inflammatory component of many neurodegenerative diseases. This process is not well understood and it seems at baseline that the BBB could still have residual effects in preventing the mixing of CSF and blood (Blennow et al., 1990; Berzin et al., 2000; Begley and Brightman, 2003; Zipser et al., 2007; Cheng et al., 2013). Other sources of miRNA such as urine, milk, and saliva may be less invasive and cost-effective (Cheng et al., 2013; Sheinerman and Umansky, 2013). However, they may contain more contaminants. Urine, for example, tends to be very rich in cells and can contain microorganisms in addition to renal, hematologic, and other cellular debris, providing insufficient or unsatisfactory data (Cheng et al., 2013).

## Discussion

### CNS degenerative diseases

Abnormal CSF miRNA profiles could diagnose various CNS disorders, such as AD. Cogswell et al. was one of the earliest to discover that CSF miRNA profiles could differentiate AD from control patients. CSF was isolated from 20 donors, including Braak Stage 5 (severe AD) and Braak Stage 1 (nondemented controls). MiR-27a, miR-125b, miR-146, and 57 others were significantly upregulated or downregulated in the Braak 5 population relative to the Braak 1 population. These dysregulated miRNAs were found to be involved in immune cell function, T cell signaling, and T cell activation (Cogswell et al., 2008).

Recent studies have validated the use of CSF miRNA in AD detection. Galimberti et al. studied 22 AD patients and 18 Non-inflammatory neurological disease controls (NINDC). In a confirmatory study, they evaluated 15 AD and 12 NINDC patients. They found significantly decreased levels of CSF miR-125b and miR-26b in AD patients compared to controls (Galimberti et al., 2014). Likewise, miR-802, which is located in the RUNX1 gene (greatly overexpressed in AD) has been shown to be correlated with early AD (van Harten et al., 2011).

Galimberti et al. reported a negative correlation of CSF miR-26b with total and phosphorylated Tau (T-Tau and P-Tau) levels in AD patients (Galimberti et al., 2014). Sala et al. analyzed 8 miRNAs from 2 age-matched cohorts and found 1-fold lower CSF miR-27a-3p expression and 1-fold higher miR-216a-5p and miR-210 expression in AD patients. Levels of these miRNA were also correlated with T-tau and P-tau values (Sala Frigerio et al., 2013). It is encouraging that many of these CSF miRNAs correlate with proteins that are virtually pathognomonic for AD such as Tau and Aβ. The dysregulation of miR-29b and miR-125b were further studied in the CSF of 18 AD patients and 20 healthy controls using quantitative PCR. CSF miR-29a levels were increased by a factor of 2.2 in AD patients when compared to controls (Müller et al., 2015). MiR-29a is thought to play a part in the formation of BACE1, a key enzyme involved in Aβ amyloid formation (Hébert et al., 2008). AD differentiation from healthy controls using miR-29a as a biomarker had 89% sensitivity and 70% specificity. MiR-125b expression was slightly increased, with 78% sensitivity and 60% specificity in differentiating AD patients from controls (Müller et al., 2015). MiR-125b is thought to be involved in the cell growth and differentiation pathway via Hedgehog signaling (Ferretti et al., 2008).

Given new evidence for its potential role in hypoxic injury and VEGF (an angiogenic growth factor) mediated disease, miR-210 expression was quantified in 56 patients with mild cognitive impairment (MCI) and AD when compared to 42 healthy controls using quantitative PCR (Solerte et al., 2005; Liu et al., 2014; Zhu et al., 2015). Decreased miR-210 and VEGF levels were correlated with the degree of dementia and disease severity (Zhu et al., 2015).

One of the largest studies investigating Parkinson's disease (PD) and AD miRNA biomarkers was conducted in 2014 by Burgos et al. CSF miRNA profiles and post-mortem biopsies were studied in 69 patients with AD, 67 PD patients, and 78 healthy controls using next generation sequencing. Forty-one CSF miRNAs were downregulated in AD patients when compared to controls. Seventy-three percent of the dysregulated miRNAs have been previously linked to AD, showing reproducibility amongst miRNA studies. When compared to PD patients, only miR-32-5p was found to be unique to AD. Seventeen miRNAs were found to be dysregulated and 14 downregulated in the CSF of PD patients compared to controls. Additionally, decreased CSF expression of miR-9-3p and miR-708-3p correlated with an increased Braak score. MiR-101-3p was also linked to amyloid precursor protein (APP) expression and Tau phosphorylation. Furthermore, Lewy body pathology progression was found to be linked to higher miR-34a-5p and miR-374a-5p CSF expression (Burgos et al., 2014).

In addition to AD, CSF miRNA has also been studied in amyotrophic lateral sclerosis (ALS). De Felice et al. examined CSF miRNA from 10 sporadic ALS (sALS) patients and 10 controls using qRT-PCR. Several CSF miRNA were differentially expressed in sALS patients relative to controls, including miR-338-3p (De Felice et al., 2014). MiR-388-3p is of particular interest because it had been previously shown to be upregulated in frontal cortex tissues, blood, leukocytes, serum, and spinal cord tissues (Shioya et al., 2010; De Felice et al., 2014; Cloutier et al., 2015). In addition, MiR-338-3p has been speculated to be involved in many neuronal functions, including neuroblast and mature neuron apoptosis as well as neuronal metabolic pathway regulation (Aschrafi et al., 2008; Ragusa et al., 2010). Elevation of other miRNAs, such as CSF miR-27b, miR-146a, and miR-532-3p, cannot only be used to recognize ALS but also enable the differentiation of ALS from multiple sclerosis (Cloutier et al., 2015).

Freischmidt et al. also found a strong link between dysregulated miRNA and TDP-43 in ALS patients. Freischmidt analyzed the CSF miRNA from 22 sALS patients and 24 control subjects and found that 5 out of 9 miRNA associated with TDP-43 were dysregulated in the CSF of ALS patients. MiR-132-5p, miR-132-3p, miR-143-3p were upregulated while miR-143-5p and miR-574-5p were downregulated (Freischmidt et al., 2013). TDP-43 is a central player in ALS pathogenesis and therefore its linkage with miRNAs makes miRNA even more credible as a biomarker.

Lastly, CSF miRNA profile has been examined in multiple sclerosis (MS). CSF miRNA expression was studied in 53 MS patients and 39 controls. MiR-181c and miR-633 were upregulated and miR-922 was downregulated in MS patients when compared to controls. When combined, miR-633 and miR-922 was 88% sensitive and 69% specific in MS diagnosis. Additionally, miR-181c and miR-633 could be used to differentiate relapsing-remitting (RRMS) and secondary progressive MS (SPMS) courses with a specificity of 82% specificity and sensitivity of 69%. These miRNA levels were increased in RRMS when compared to SPMS, but were expressed at higher levels in all MS cases compared to controls (Haghikia et al., 2012; See Table 1).

**Table 1:** **Potential microRNA biomarkers identified in plasma and Cerebrospinal Fluid (CSF) in different neurodegenerative diseases**.

**Disorder**	**Increased**	**Decreased**
Alzheimer's disease	miR-29a^a^^,^ ^b^, miR-210^c^^,^ ^d^^,^ ^e^, miR-216a^c^^,^ ^d^, miR-802^f^	miR-9^g^, miR-26b^a^^,^ ^h^, miR-27a^a^^,^ ^c^^,^ ^d^, miR-125b^a^^,^ ^b^^,^ ^h^
Parkinson's disease		Overall downregulation
Amyotrophic lateral sclerosis	miR-338-3p^i^ 143-5p^j^	132-3p, 132-5p, 143-3p, let-7b^j^^,^ ^k^
Multiple sclerosis	miR-181c, miR-633	miR-633 + miR-922

a *Kiko et al. (2014)*;

b *Müller et al. (2015)*;

c *Cogswell et al. (2008)*;

d *Sala Frigerio et al. (2013)*;

e *Zhu et al. (2015)*;

f *van Harten et al. (2011)*;

g *Burgos et al. (2014)*;

h *Galimberti et al. (2014)*;

i *De Felice et al. (2014)*;

j *Freischmidt et al. (2013)*;

k *Cloutier et al. (2015)*.

### Other conditions

Beyond neurodegenerative disorders, CSF miRNA profiles have been explored in CNS malignancies. In a study examining 23 primary CNS lymphoma (PCNSL) patients and 30 controls and a follow-up with 16 additional PCNSL patients, Baraniskin et al. found that miR-21, miR-19b, and miR-92 were among the most highly upregulated miRNA in PCNSL (Baraniskin et al., 2011). These miRNA had a95.7–97.4% sensitivity and 96.7% specificity for PCNSL and could also differentiate PCNSL from inflammatory CNS disorders. In addition, these miRNA were found to correlate with tumor volume on MRI, as well as disease progression, remission, and relapse in response to chemotherapy (Baraniskin et al., 2011, 2012a). Of the 3 upregulated miRNA, miR-21 was the most highly elevated, with a relative expression level of 60.0 in PCNSL and 3.8 in controls (Baraniskin et al., 2011). MiR-21 is upregulated in a variety of CNS tumors, possibly because of its role in targeting tumor suppressors Acidic Nuclear Phosphoprotein 32 Family, Member A (ANP32A) and SWI/SNF-related, Matrix-associated, Actin-dependent Regulator Chromatin group A (SMARCA4) (Akers et al., 2013).

CSF miRNA profiling has also been explored in gliomas and glioblastomas. Like PCNSL, gliomas are characterized by miR-21 over-expression, with a 85–87% sensitivity and 93–100% specificity in glioblastoma detection (Gabriely et al., 2008; Baraniskin et al., 2012a; Akers et al., 2013). Akers et al. reported a decreased miR-21 level of more than 50-fold in a surgical patient as compared to pre-surgery level, suggesting that miR-21 can potentially be used to determine treatment efficacy (Akers et al., 2013). Furthermore, low expression of MiR-10b was found in normal individuals, and high expression was noted in roughly 90% of glioma subjects. Additionally, miR-10b correlates well with erlotinib treatment, CSF cytology, and MRI results (Teplyuk et al., 2012). Finally, miR-15b, can detect glioma with a 90% sensitivity and 94.9% specificity alone or a 90% sensitivity and 100% specificity when measured along with miR-21 (Baraniskin et al., 2012b).

In addition to cancers and neurodegenerative diseases, brain injuries and infections can also potentially be detected by CSF miRNA profiling. Traumatic brain injuries (TBI), for example, are associated with elevated miR-let-7i and miR-451, which are thought to produce TBI-associated damages through the action of cytokines S100b and UCH L1. TBIs are also associated with decreased miR-9 levels and higher overall CSF miRNA levels (Balakathiresan et al., 2012; Patz et al., 2013). HIV encephalitis (HIVE) patients, while they do not exhibit any higher overall CSF miRNA, do show increased levels of miR-937, miR-598 and miR-595 (Pacifici et al., 2013). Moreover, stroke patients have been shown to exhibit elevated miR-let-7c and miR-221-3p (Sørensen et al., 2014). Psychiatric conditions, such as major depressive disorder (MDD), may also benefit from CSF miRNA detection Notably, miR-221-3p, miR-34a-5p, and miR-let-7d-3p levels were upregulated and miR-451 was downregulated with a sensitivity of 84.85–96.88% and specificity of 90.48–95.24% for MDD (Wan et al., 2015).

## Future directions

MiRNA CSF profiling may lead to early diagnosis of disease, allowing for early prevention implementation and timely treatment of many disorders. For example, diffuse neuronal damage in TBI may be identified using miRNA biomarkers when CT scans are negative (Balakathiresan et al., 2012; Rao et al., 2013). MiRNA may also guide prognosis and help determine the aggressiveness of the required treatment, such as suicide risk-stratification in MDD or tumor staging (Baraniskin et al., 2012a; Wan et al., 2015). Longitudinal miRNA examination may allow for tracking of disease progression and treatment efficacy (Baraniskin et al., 2012a). These, and many other applications of miRNA CSF profiling have yet to be fully explored.

Altered miRNA expression has been noted in the serum and brain tissue samples of patients with frontotemporal dementia, progressive supranuclear palsy, bipolar affective disorder, addiction/substance abuse, anxiety disorders, autism, Tourette's syndrome, postoperative delirium, oligodendroma, astrocytoma and medulloblastoma. Specific miRNA have also been implicated in some of these disease processes, and show promise for further examination as CSF biomarkers (De Smaele et al., 2010; Chan and Kocerha, 2012; Stoicea et al., 2013; Kolshus et al., 2014; Ruggeri et al., 2014). Additionally, miRNA CSF have been studied in schizophrenia, but the small sample size precluded comparison between diseased and control CSF levels (Gallego et al., 2012).

A meta-analysis concluded that the strongest singular CSF miRNA biomarker for glioma, miR-21, achieved 82% sensitivity and 94% specificity. However, pooled results of all miRNA studied and taken as a diagnostic panel, had a sensitivity of 91% sensitivity and specificity of 94% (Qu et al., 2015). Additionally, miRNA combinations are sometimes required to differentiate one particular CNS disease from another since miRNAs have such widespread action. While increased miR-21 and miR-10b are indicative of various types of brain cancer, relatively increased miR-200 signifies metastasis over primary tumor. When 7 miRNAs (miR-10b, miR-21, miR-125b, miR-141, and several miR-200s) were studied as a panel, diagnostic probability was higher at 99% than single biomarkers (Teplyuk et al., 2012).

Many studies searched miRNA gene banks to connect miRNAs with disease-related proteins. Matches either supported previously hypothesized disease processes or proposed new processes (Gabriely et al., 2008; Hébert et al., 2008; Robertus et al., 2009; Medina et al., 2010; Balakathiresan et al., 2012; Wan et al., 2015). Continued research on miRNA and its function may further elucidate miRNA links to disease pathology and disease-causing genetic imbalances could be restored with miRNA-targeted treatment (Baraniskin et al., 2012b; Müller et al., 2015; Wan et al., 2015; Zhu et al., 2015). Oved et al. concluded that treatment of human cell tissue lines with paroxetine decreased miR-221 and miR-222 (Oved et al., 2013). Additionally, miravirsen, an anti-miR-122 treatment, is currently in Phase 2 clinical trials for the treatment of chronic hepatitis C (Kolshus et al., 2014).

Although this review presents strong evidence for the clinical importance of CSF biomarkers, there are a few limitations. Among the various studies, there was significant control group variation in number, as CSF can be difficult to obtain in healthy individuals (Baraniskin et al., 2011, 2012b; Haghikia et al., 2012; Sørensen et al., 2014; Müller et al., 2015; Zhu et al., 2015). Also, there isn't a control group data base for miRNA expression that would normalize background variability for disease profile comparison. Additionally, most of the studies had small sample sizes, some with as few as 6 patients (Sørensen et al., 2014). Larger scale and longitudinal studies must be completed to further validate these results before clinical integration. Repetition would also help identify the most sensitive and specific miRNA biomarkers, as some studies show differing results, potentially from varying methods of analysis (Cogswell et al., 2008; Müller et al., 2015; Zhu et al., 2015). Furthermore, it should be noted that certain medications, including some anesthetics, can affect miRNA levels and potentially complicate results (Ackermann et al., 2014). Finally, lumbar puncture is not currently standard diagnostic procedure for many of these conditions, including PCNSL, glioma, TBI and depression (Baraniskin et al., 2011, 2012b; Balakathiresan et al., 2012; Wan et al., 2015).

Additionally, various methods are used in miRNA acquisition, extraction, amplification, processing, quantification, normalization and statistical analysis. This is even further complicated since miRNA's stability allows analysis from preserved, frozen, and fresh tissues and fluids (Xi et al., 2007; Weber et al., 2010). However, sample quality, time elapsed since collection, and storage methods can all affect results (De Smaele et al., 2010). The various extraction methods and reagents used yield different miRNA levels and profiles (Eldh et al., 2012). Further discrepancies may result from the multitude of data processing software and data normalization techniques available for use (Meyer et al., 2010; Garber et al., 2011).

Although new techniques are being developed, there are three major approaches to miRNA-specific quantification: quantitative reverse transcription polymerase chain reaction (qrPCR), hybridization-based methods using arrays, and high-throughput sequencing or next generation sequencing (nextgen). Each technique has its own strengths and weaknesses. QrPCR provides the most accurate absolute miRNA quantification and is already widely used in DNA and RNA processing. However, reaction conditions can differ between machines and samples, and it is not ideal for novel miRNA identification. This is the least expensive miRNA analysis method (Pritchard et al., 2012). Microarrays of fluorescent probes or beads are best used to compare miRNA levels between samples, but are less accurate, sensitive, and reliable for absolute quantification because non-identical sequences can hybridize (Pritchard et al., 2012). This well-established method requires a larger amount of starting miRNA (De Smaele et al., 2010). Nextgen is a promising new technique that measures relative miRNA expression, as opposed to direct absolute quantification. It can accurately differentiate similar miRNAs and identify novel miRNA. Nextgen quantification is also able to yield a massive amount of data for computation. Methods other than nextgen-like techniques require previous knowledge about the miRNA in question, so results can be limited and biased. As a new technology, nextgen is the most expensive miRNA analysis method (Meyer et al., 2010)

## Conclusion

Although a newly emerging field, CSF miRNA sampling and expression profiling show promise as a noninvasive CNS biomarker. MiRNAs exhibit widespread genetic action, playing a role in many cellular functions, and have been linked to pathological processes. MiRNA changes are often detectable prior to their downstream protein expression effects. They are protected and stable in various body fluids and tissues, making them a valuable biomarker. While serum miRNA can be a useful biomarker in various other diseases, the BBB might preclude its use in the diagnosis of neurological disorders. CSF, a promising alternative, is a relatively cell- and microorganism-free fluid.

The CNS is the least surgically accessible body system, making diagnosis of disorders invasive, costly, and potentially dangerous. Lumbar puncture is already part of the diagnostic workup of many neurodegenerative diseases including AD and PD. That being said, miRNA CSF profiling would be relatively easy to integrate into the management of these diseases. However, the current diagnostic workup of psychiatric disorders and brain cancers relies primarily on clinical examination and invasive brain biopsy. While currently, miRNA CSF profiling focuses mainly on disease identification, there are numerous other applications including prognosis, course tracking, and potential treatment of CNS diseases. Alongside patient history, physical exam, and clinical testing, miRNA profiles have the potential to contribute positively to patient care and outcomes.

## Author contributions

The authors declare equal contribution in the writing, editing and final version of the present manuscript.

### Conflict of interest statement

The authors declare that the research was conducted in the absence of any commercial or financial relationships that could be construed as a potential conflict of interest.
